# Supplementary material for: Identification of spatially-resolved markers of malignant transformation in Intraductal Papillary Mucinous Neoplasms
Source: Nat Commun. 2024 Mar 29;15:2764. doi: 10.1038/s41467-024-46994-2 (PMC10980816; doi:10.1038/s41467-024-46994-2)
Supplement: Supplementary file 1 — Supplementary Information [file 41467_2024_46994_MOESM1_ESM.pdf]

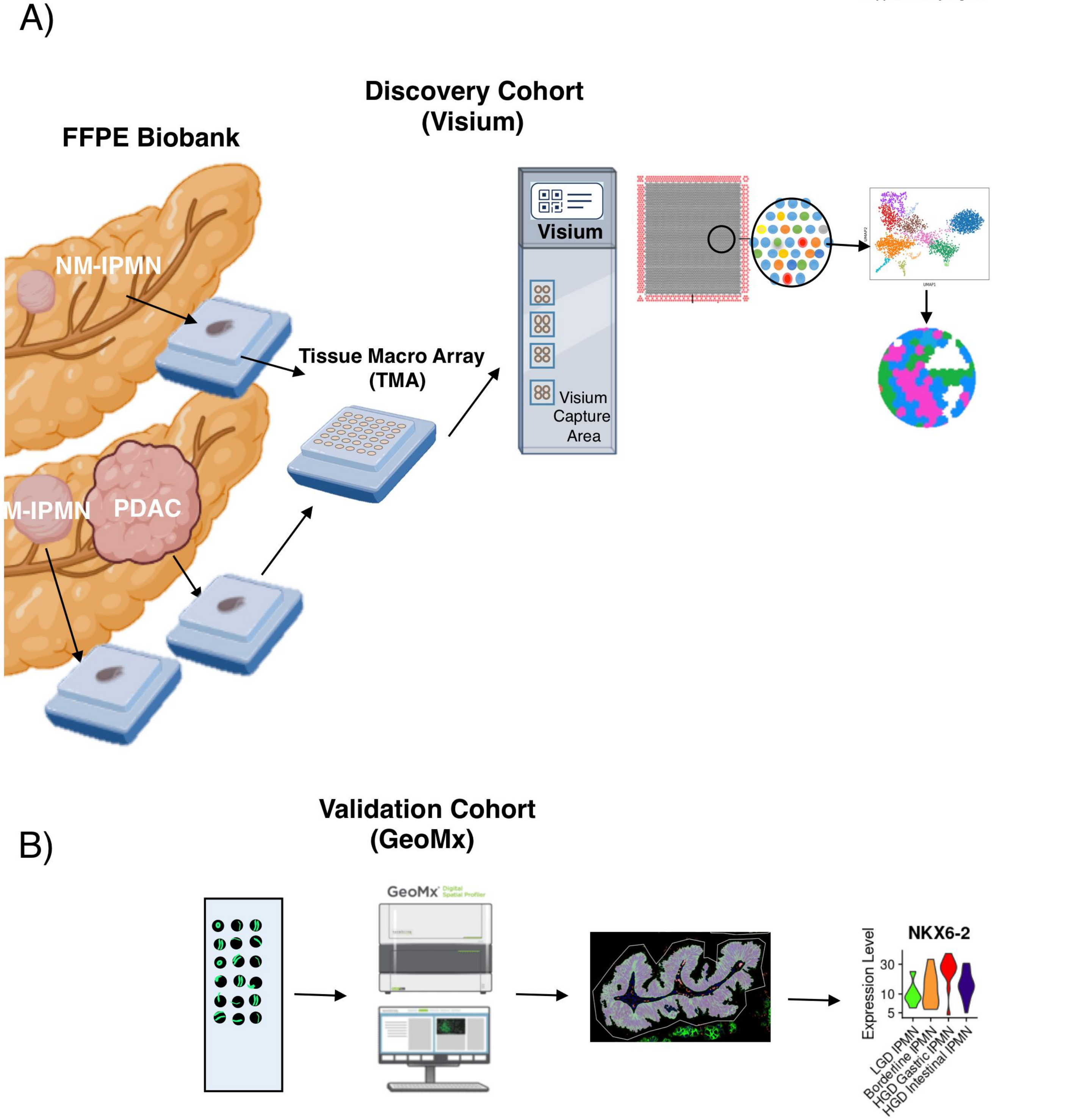

Supplementary Figure S1. Workflow for Spatial Transcriptomics Analysis. A) Discovery cohort was analyzed with Visium spatial transcriptomics. FFPE IPMN samples were gathered from our institution and four TMAs were built with each 1,5 mm core representing one IPMN/PDAC sample. Each TMA was included in one capture are of a Visium Slide and processed following the standard recommendation. After sequencing the Visium data was analyzed with Seurat R package and spatial clusters were identified. B) Validation Cohort consisting of two TMAs were analyzed with GeoMX. Each TMA slide was stained by immunofluorescence with GEOMX morphology markers for PanCK and CD45. ROI were selected a segmented to isolate only the PanCK positive IPMN region. After sequencing GeoMx data was analyzed with Seurat. The picture was created with Biorender.com. Abbreviations: Low-Grade-Dysplasia, LGD; High-Grade-Dysplasia, HGD.

TMA	Grading	Cyst Main Morphology§	Duct type	Grade of Dysplasia	Radius (mm)	Sample Annotation
1	low-grade	Gastric morphology	branch	low-grade	35	LGD
1	low-grade	Gastric morphology	main	low grade	45	LGD
1	high-grade	Gastric morphology	branch	high-grade	45	HGD Gastric
2	low-grade	Gastric morphology	branch	low grade	45	LGD
2	low-grade	Gastric morphology	main	intermediate-grade	15	Borderline
2	high-grade	Gastric morphology	branch	high-grade	40	HGD Gastric
3	high-grade	Intestinal morphology	main	high-grade	25	HGD Intestinal
3	high-grade	Intestinal morphology	main	high-grade	30	HGD Intestinal
3	high-grade	Pancreatobiliary morphology	main	high-grade	60	HGD Pancreatobiliary
3	high-grade	Intestinal morphology	main	high-grade	115	HGD Intestinal
4	high-grade	Gastric morphology	main	high-grade	40	HGD Gastric
4	high-grade	Gastric morphology	branch	high-grade	70	HGD Gastric
4	high-grade	Gastric morphology	branch	high-grade	35	HGD Gastric
4	high-grade	Gastric morphology	branch	high-grade	37	HGD Gastric

§ The morphology refers to the prevalent morphology identified by microscopical evaluation of multiple sections of the same IPMN sample.

Supplementary Table S1. Main macroscopic and histological features of Discovery cohort IPMN samples. The table show the main histological characteristics of the IPMN samples included in the discovery cohort. Abbreviations: Low-Grade-Dysplasia, LGD High-Grade-Dysplasia, HGD.

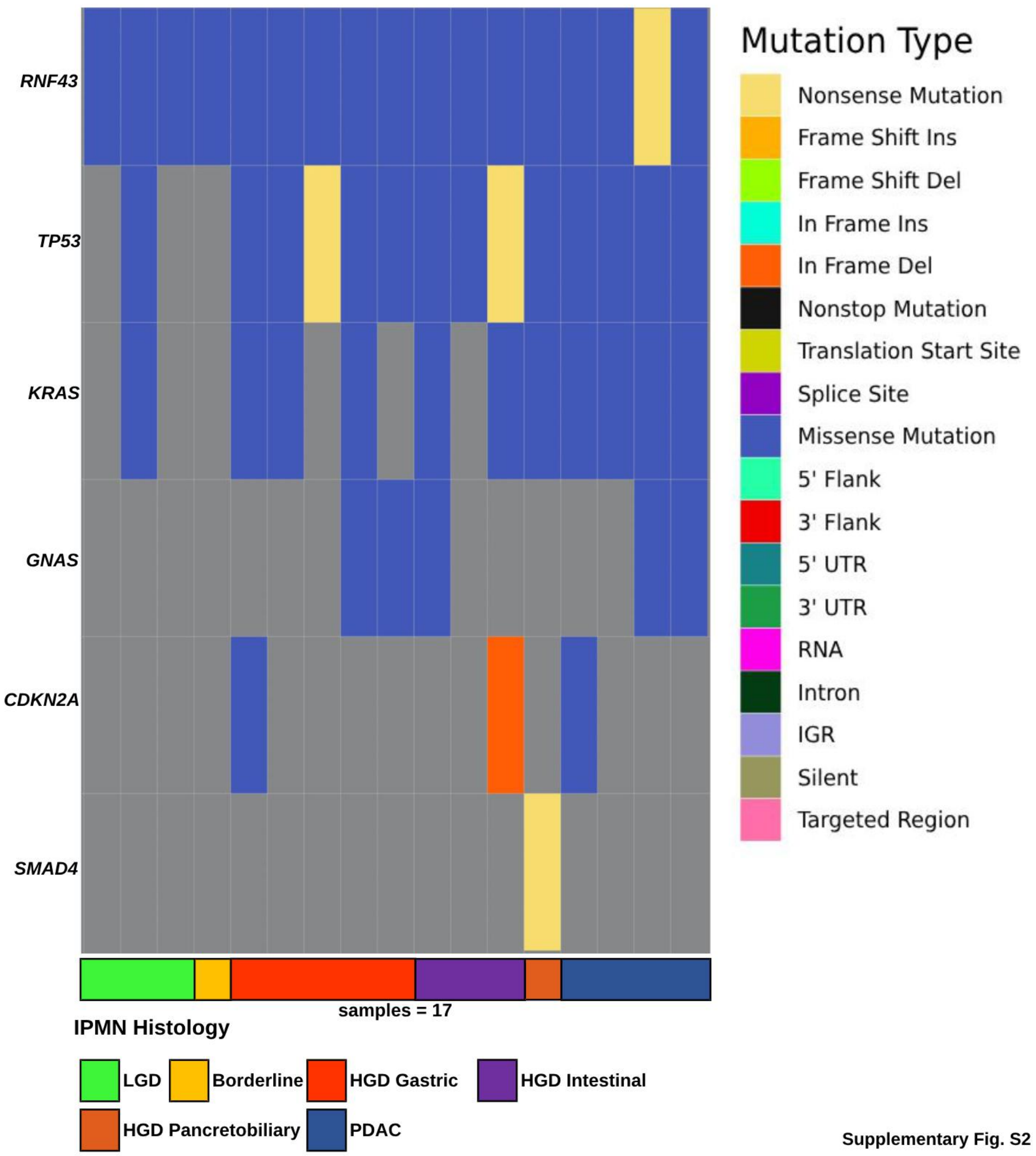

Supplementary Figure S2. TruSight 500 genomic profiling. Oncoplot showing the pattern of the most recurrent IPMN mutations in the Visium cohort IPMN samples. Abbreviations: Low-Grade-Dysplasia, LGD; High-Grade-Dysplasia, HGD; Pancreatic Ductal Adenocarcinoma, PDAC; Insertion, Ins; Deletion, Del; UnTranslated Region, UTR; InterGenic Region, IGR.

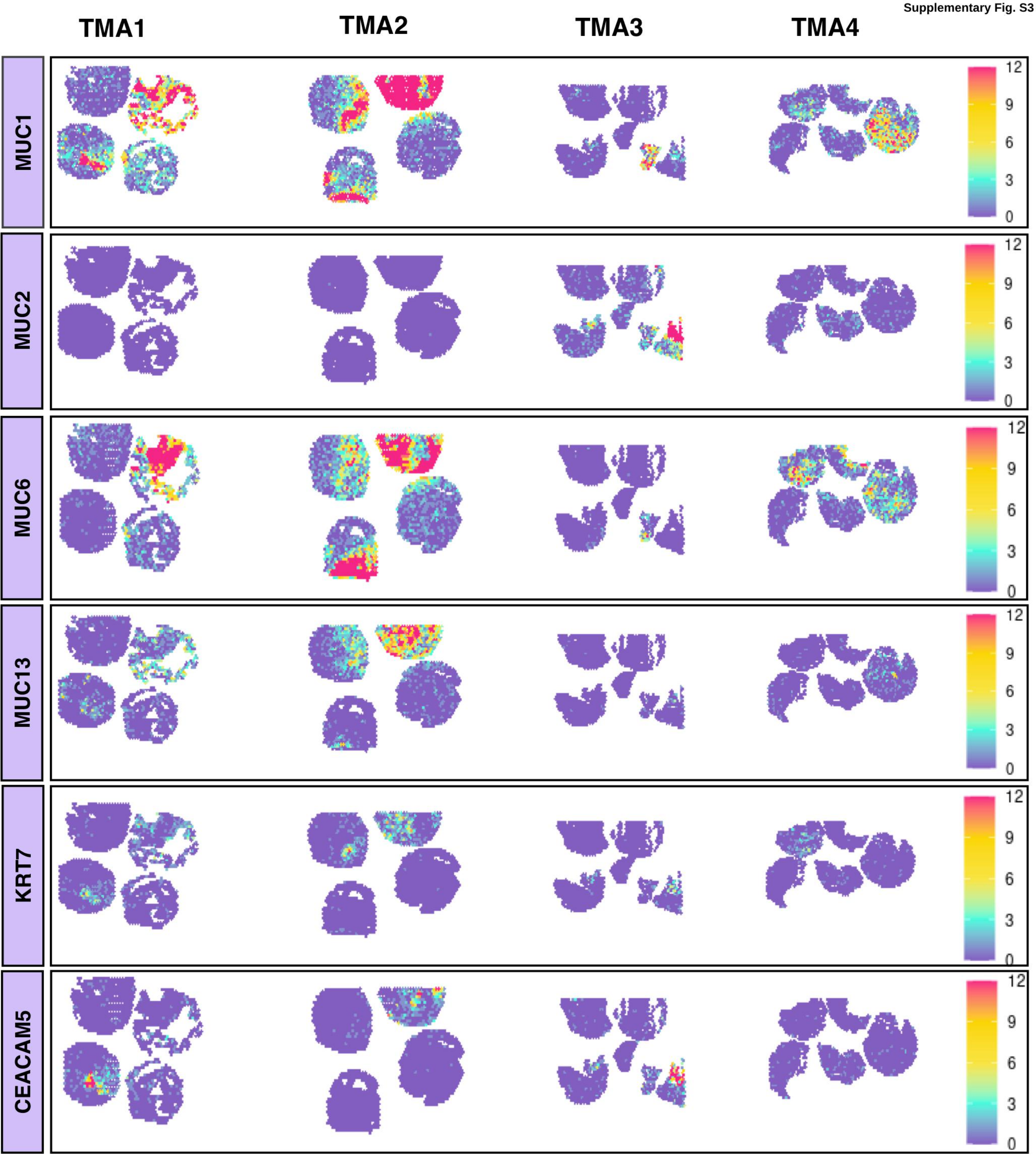

Supplementary Figure S3. Spatial expression of common Markers of IPMN. The Fig. shows the spatial expression of the common routine markers of IPMN: *MUC1, MUC2, MUC6, MUC13, KRT7, CEACAM5*. Normalized expression is showed. Abbreviations: Tissue Micro Array, TMA.

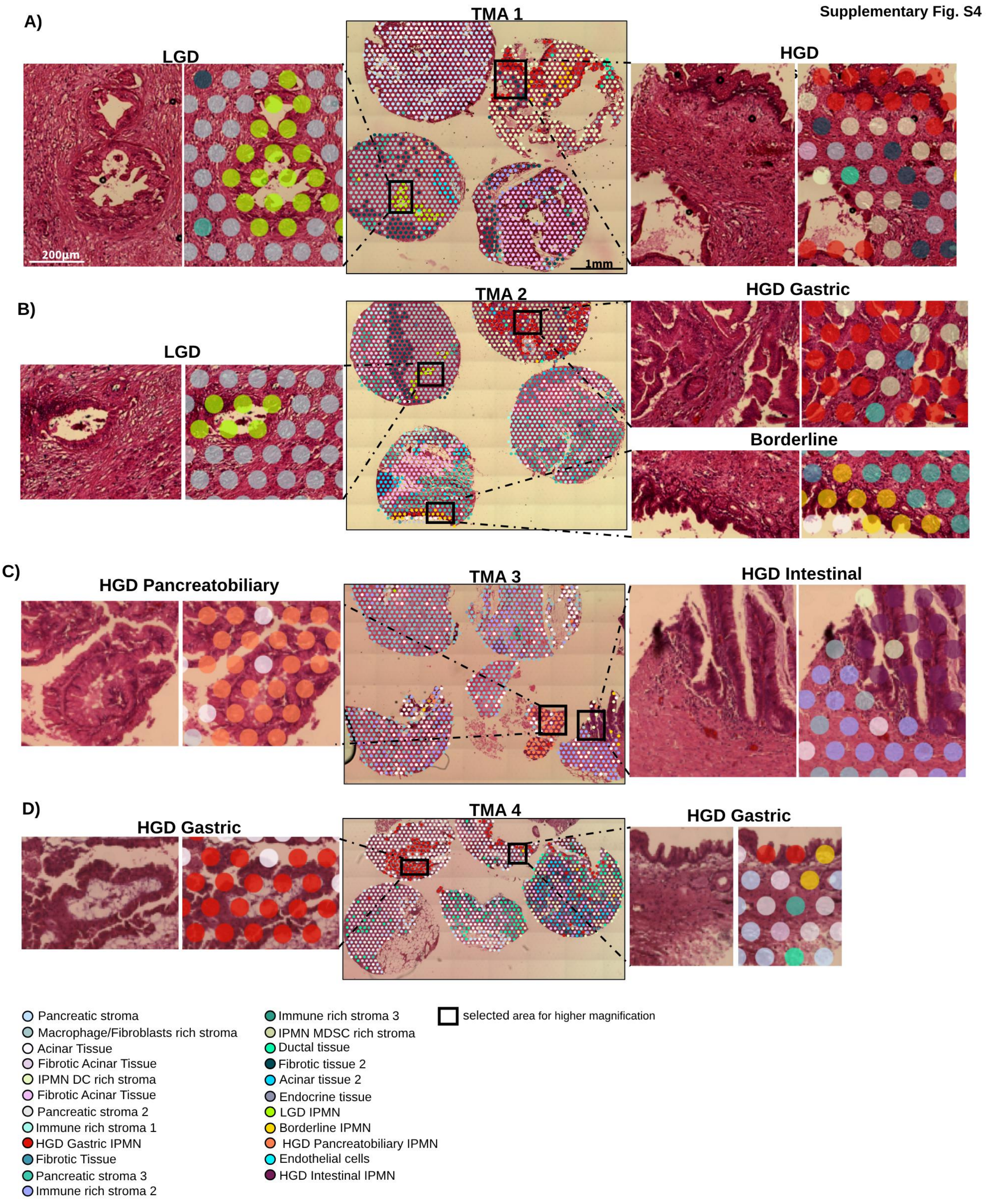

Supplementary Figure S4. Spatially-resolved clustering of Visium data. Spot level visualization of Visium clusters and correlation with histological features (H&E) of TMA1 A), TMA2 B), TMA3 C), TMA4 D). Abbreviations: Tissue Micro Array, TMA; Low-Grade-Dysplasia, LGD; High-Grade-Dysplasia, HGD.

Supplementary Note 1. Optimizing Cluster Number and Histological Associations.

To determine the optimal number of clusters and their correlation with histological features, we modulated the resolution parameters using the Findclusters() function and leiden algorithm. In our analysis, 0,85 was the best resolution parameter to prevent the occurrence of sub- or over-clustering, particularly in IPMN clusters (Figure 2).

However, comparable outcomes were achieved even when configuring parameters within a range with a ± 0.15 difference in resolution from 0.85. To emphasize the analysis, we also employed broader values (0.65 and 1.05) and extreme parameters (0.5 and 1.2). For instance, the clustering at extreme broader resolution value (0.65), displayed a discrepancy of only two stromal clusters, while all IPMN clusters remained consistent (Supplementary Figure S5).

Similarly, setting the resolution parameter to 1.05, leads to the identification of two additional stromal clusters, while the clusters for the IPMN were confirmed (Supplementary Figure S6).

Discrepancies between IPMN clusters and histological features were observed exclusively with extreme parameters (0.5 and 1.2) (Supplementary Figure S7).The use of extreme parameter (0.5) leads to the clustering of gastric and intestinal IPMNs. While the other IPMNs (LGD, Borderline, and Pancreatobiliary) continue to fall into separate clusters, confirming the different histological features of these IPMN (Supplementary Figure S8).

A clear sub-clustering becomes apparent only in gastric HGD IPMNs when using extreme parameters (1.2). At this resolution value, several subclusters are observed within the epithelium of gastric HGD IPMNs, while all the others IPMs fall into separate distinct cluster further confirming their histological features. However, the observed sub-clustering was likely due to the extreme parameter, and no statistically significant differentially expressed genes were found between the two groups using the Findmarkers function (DESeq2 method).

Supplementary Figure S5

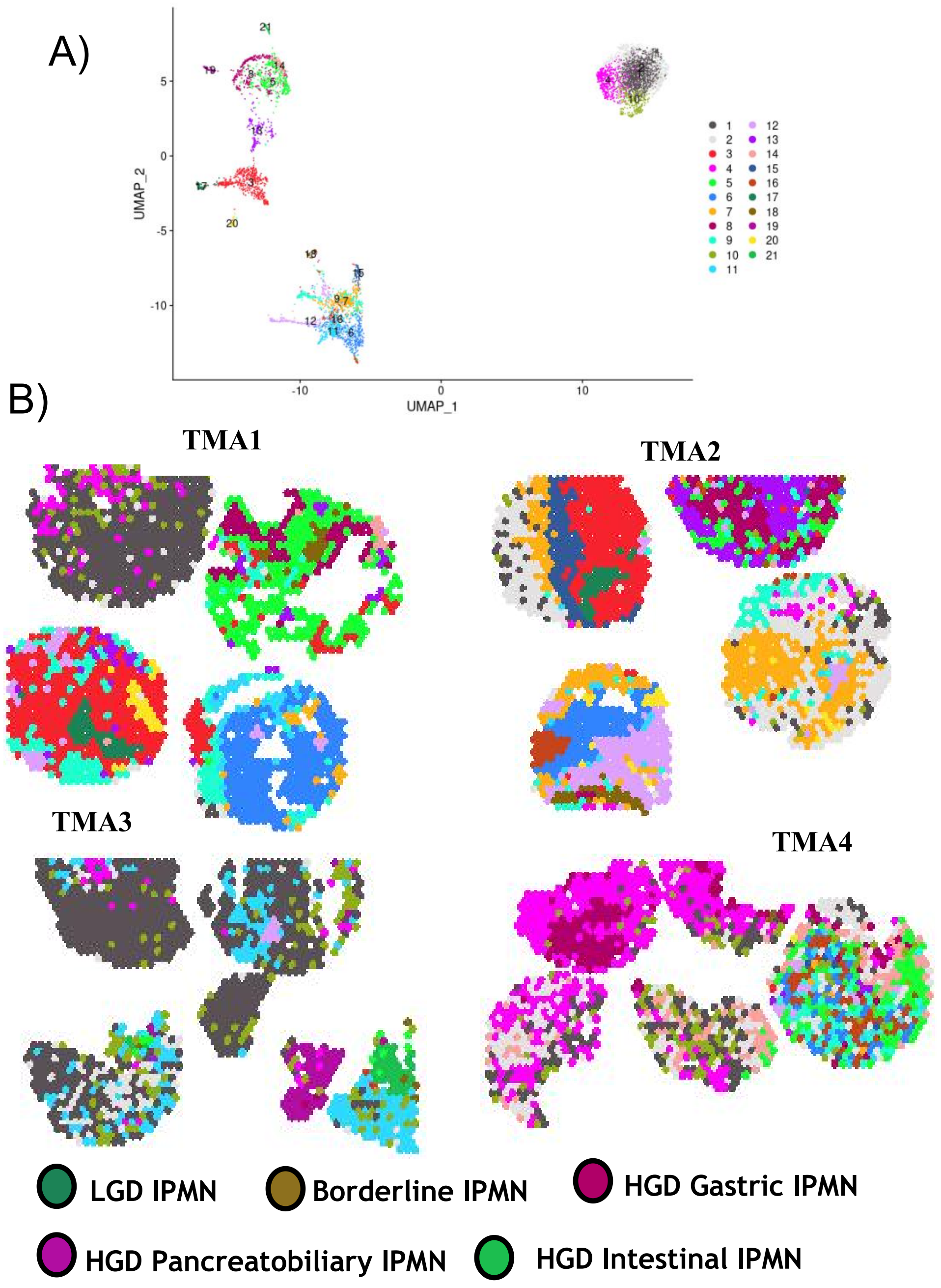

Supplementary Figure S5. Clustering using a resolution of 0.65 for leiden algorithm. A) Umap showing clustering results at 0.65 resolution. B) Spatial distribution of the clusters. Abbreviations: Low-Grade-Dysplasia, LGD; High-Grade-Dysplasia, HGD.

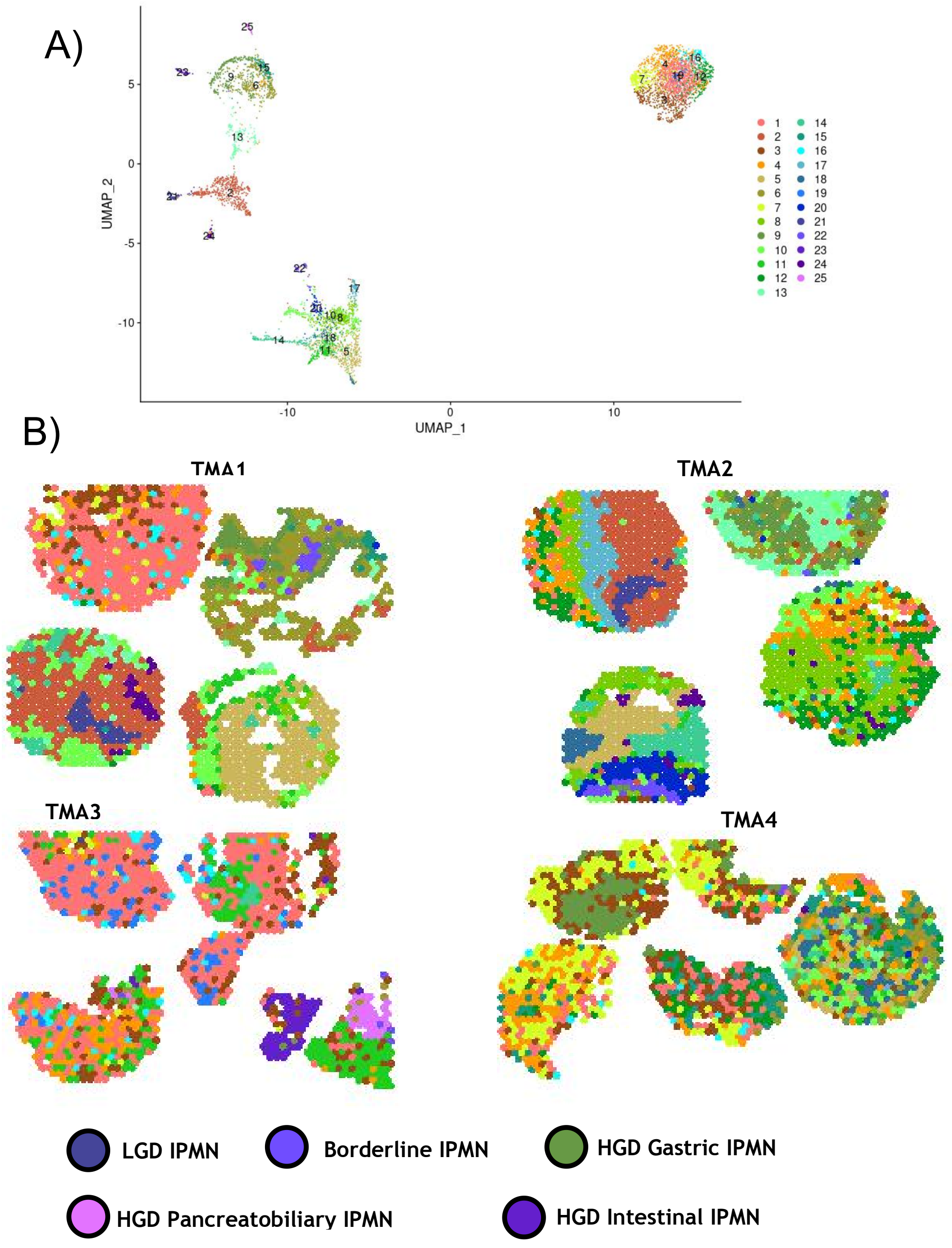

Supplementary Figure S6. Clustering using a resolution of 1.05 for leiden algorithm. A) Umap showing clustering results at 1.05 resolution. B) Spatial distribution of the clusters. Abbreviations: Low-Grade-Dysplasia, LGD; High-Grade-Dysplasia, HGD.

Supplementary Figure S7

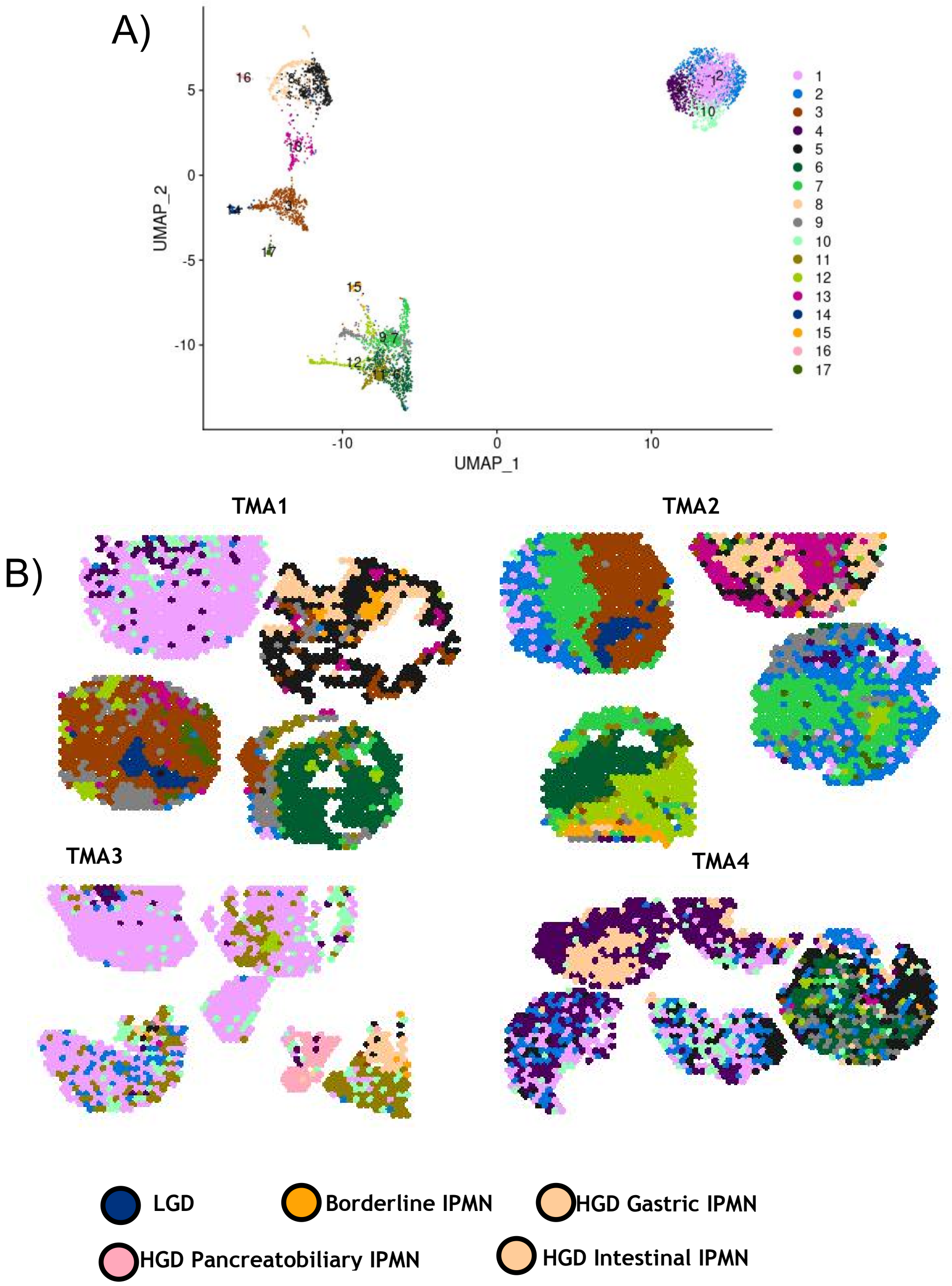

Supplementary Figure S7. Clustering using a resolution of 0.5 for leiden algorithm. A) Umap showing clustering results at 0.55 resolution. B) Spatial distribution of the clusters. Abbreviations: Low-Grade-Dysplasia, LGD; High-Grade-Dysplasia,

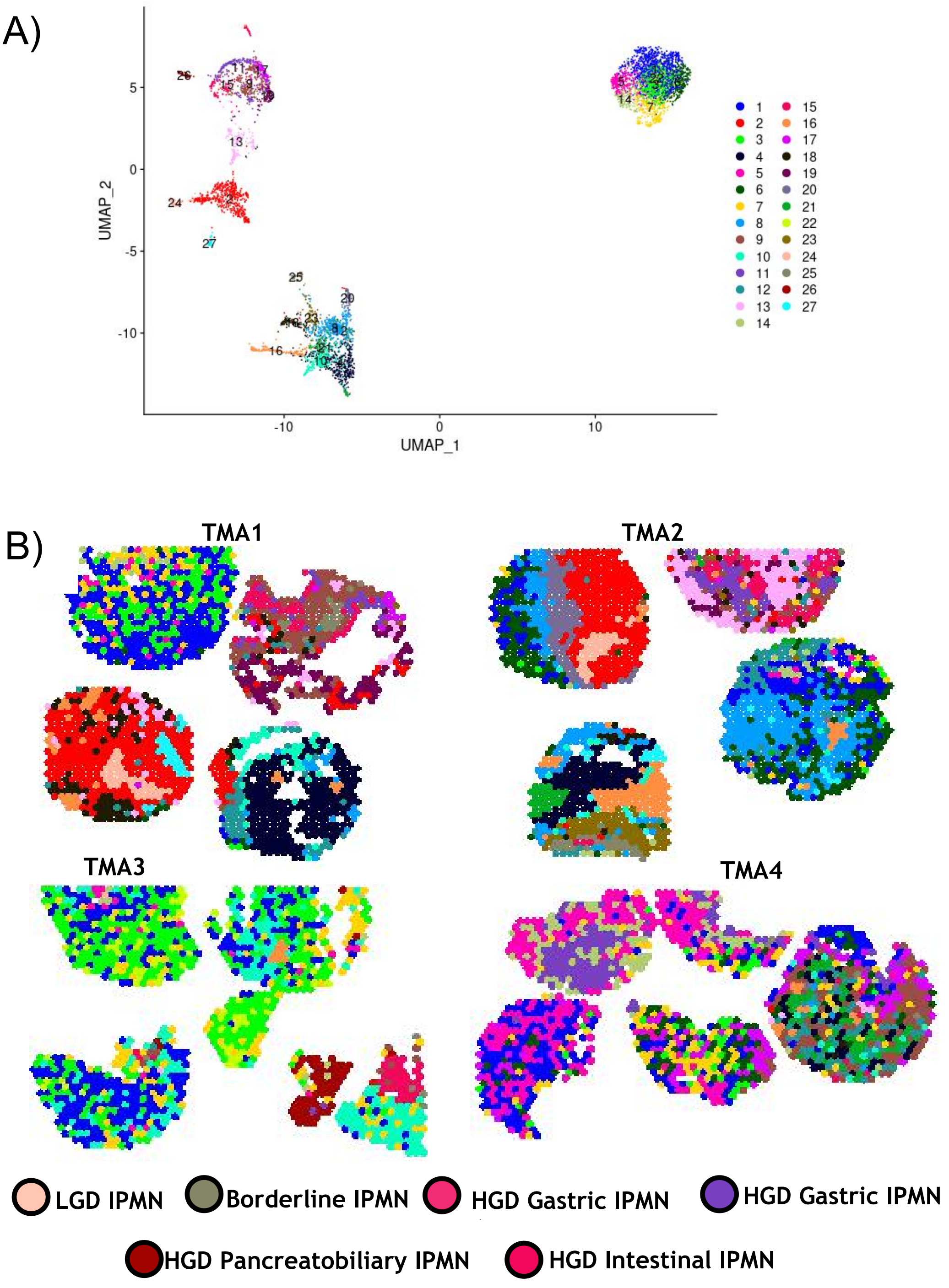

Supplementary Figure S8. Clustering using a resolution of 1.2 for leiden algorithm. A) Umap showing clustering results at 1.2 resolution. B) Spatial distribution of the clusters. Abbreviations: Low-Grade-Dysplasia, LGD; High-Grade-Dysplasia, HGD.

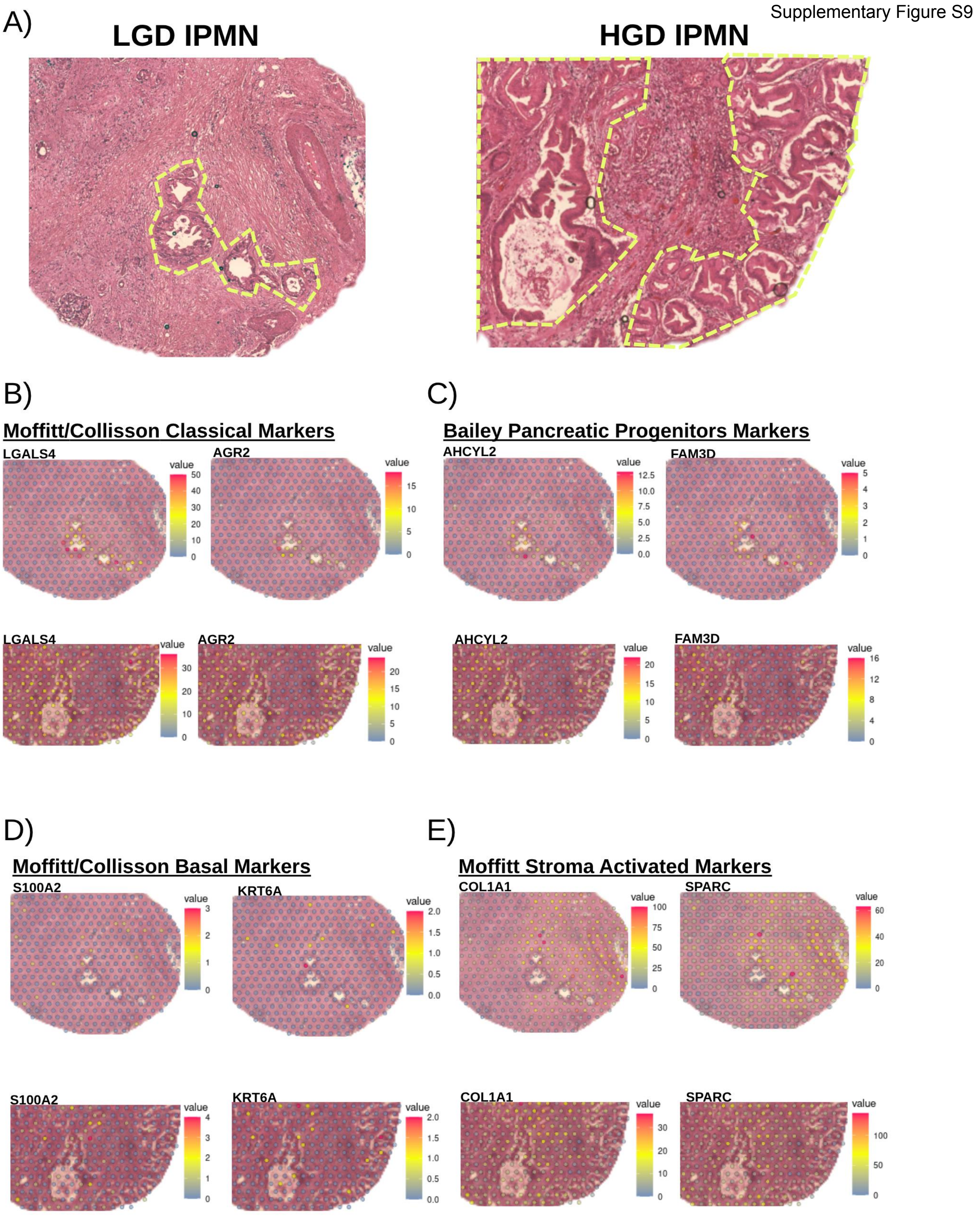

Supplementary Figure S9. Visium data spot level visualization of molecular markers of PDAC. A) High resolution pathological annotation of representative LGD and HGD IPMN (H&E staining). B) and C) The Moffitt/Collisson classical and Bailey Pancreatic Progenitor markers were specifically expressed by both LGD and HGD IPMN. D) The basal markers *S100A2* and *KRT6A* were absent. E) The Moffitt stroma activated markers were instead abundant in IPMN-surrounding stroma but not in the epithelial cells. Abbreviations: Low-Grade-Dysplasia, LGD; High-Grade-Dysplasia, HGD.

Supplementary Figure S10

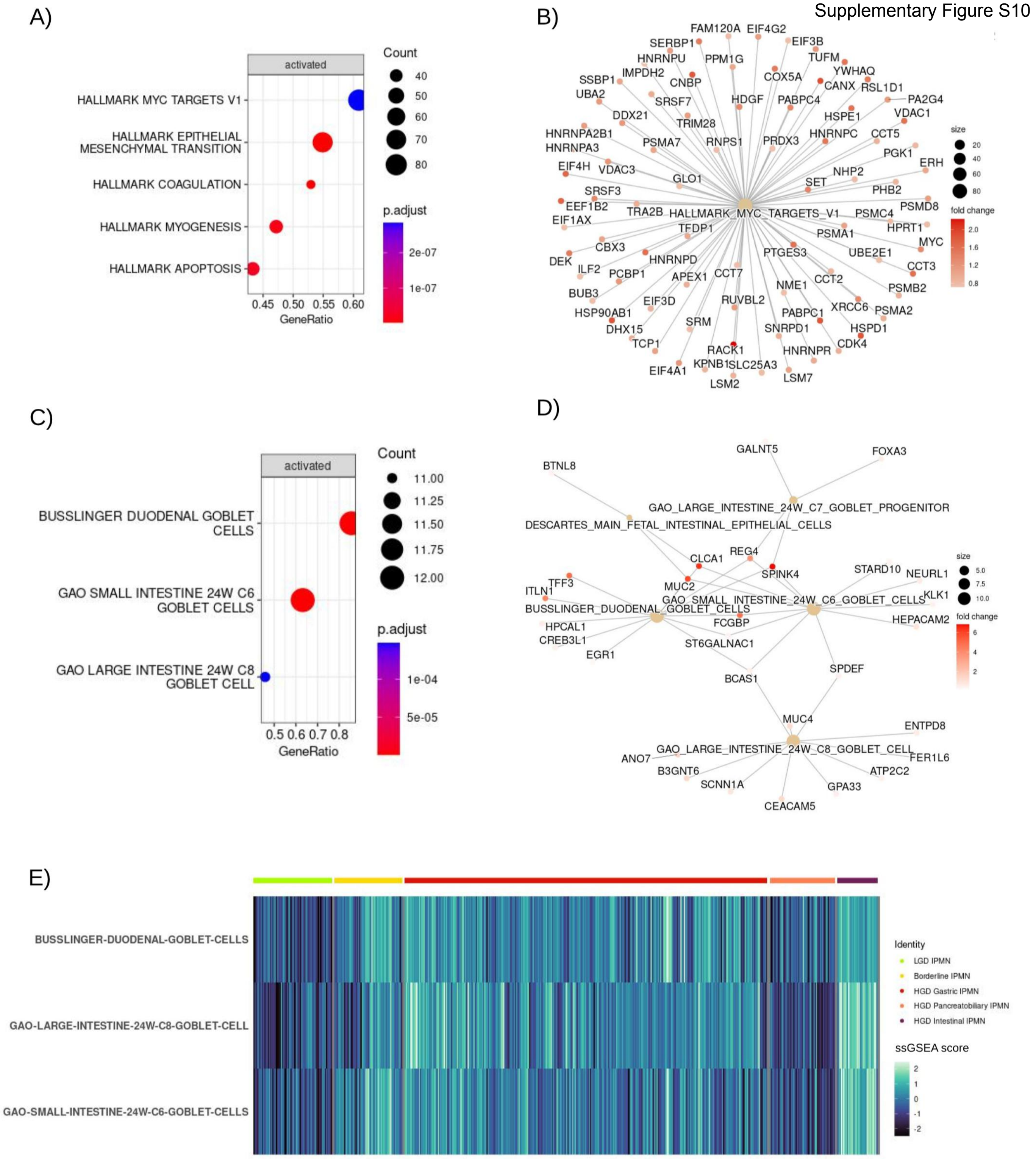

Supplementary Figure S10. GSEA results of the comparison between HGD Intestinal IPMN and LGD IPMN. A) Top five Hallmark Cancer pathways activated in HGD Intestinal IPMN. The dot size represents the number of genes upregulated. The dot size represents the number of genes overexpressed or downregulated. Two-tailed GSEA corrected for multiple comparisons with FDR <0.05. B) Network plot showing the upregulation of MYC targets genes in HGD intestinal in respect to LGD IPMN. C) Cell-type signatures overexpressed in HGD Intestinal IPMN. D) Network plot showing the overexpression of intestinal markers in HGD Intestinal IPMN. E) Heatmap showing ssGSEA for intestinal cell signatures in all IPMN clusters. Abbreviations: Low-Grade-Dysplasia, LGD; High-Grade-Dysplasia, HGD; False Discovery Rate; FDR. Source data are provided as a Source Data file.

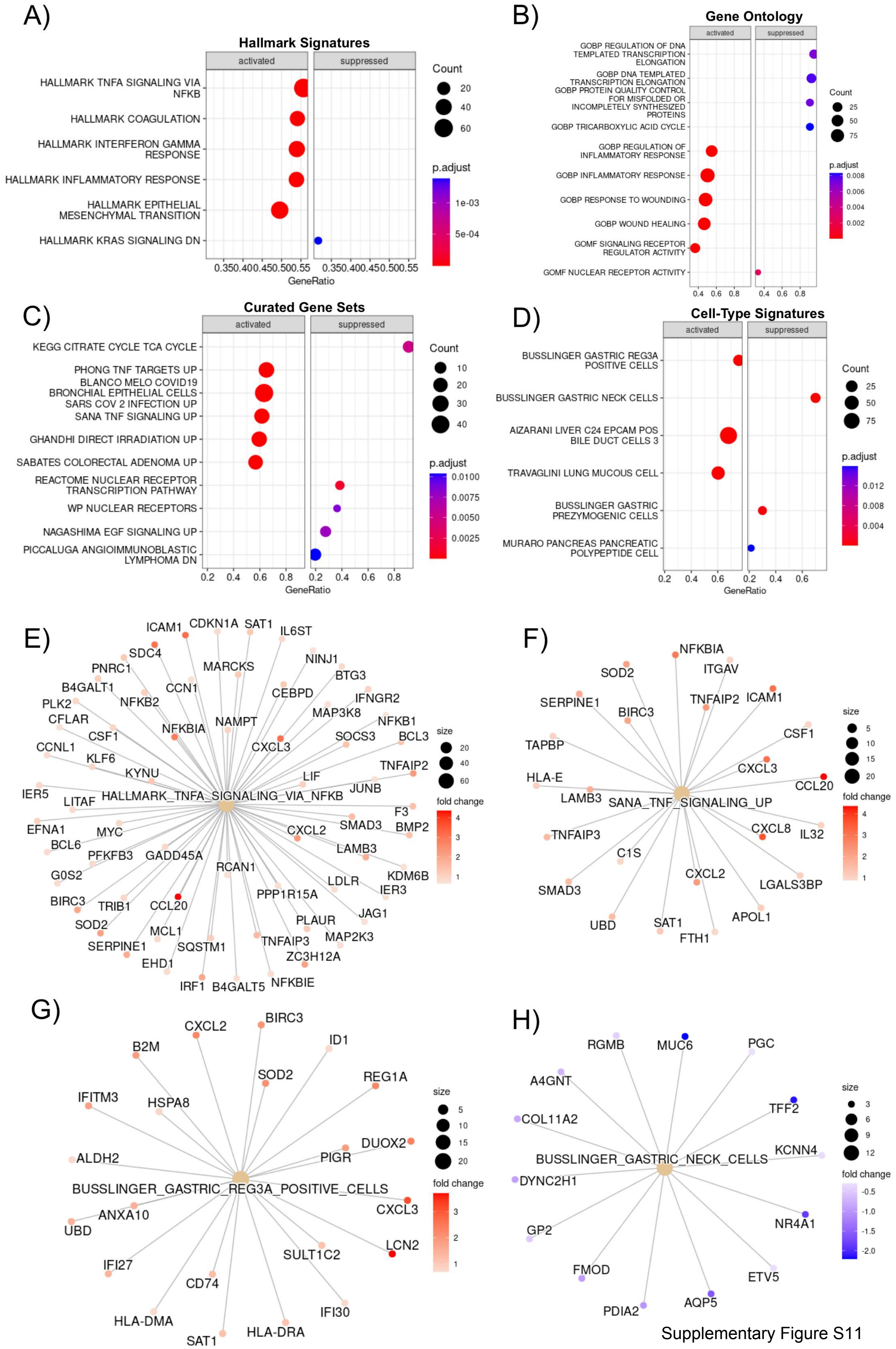

Supplementary Figure S11

Supplementary Figure S11. GSEA results of the comparison between HGD Gastric IPMN and Borderline IPMN. A) Top Hallmark Cancer pathways activated in HGD Gastric IPMN. The dot size represents the number of genes upregulated B) Top five activated and suppressed gene ontology signatures activated or suppressed in HGD Gastric IPMN. The dot size represents the number of genes overexpressed or downregulated. Two-tailed GSEA corrected for multiple comparisons with FDR <0.05. C) Top five activated and suppressed curated gene set activated or suppressed in HGD Gastric IPMN. The dot size represents the number of genes overexpressed or downregulated. D) Top activated and suppressed cell type signatures activated or suppressed in HGD Gastric IPMN. The dot size represents the number of genes overexpressed or downregulated. E) and F) Network plot showing the TNFα signalling associated genes upregulated in HGD Gastric IPMN. Network plot showing the network of gene that are regulated by Myc. G) and H) Network plots showing the expression of the cell type specific signatures. Abbreviations: Low-Grade-Dysplasia, LGD; High-Grade-Dysplasia, HGD; False Discovery Rate; FDR.Source data are provided as a Source Data file.

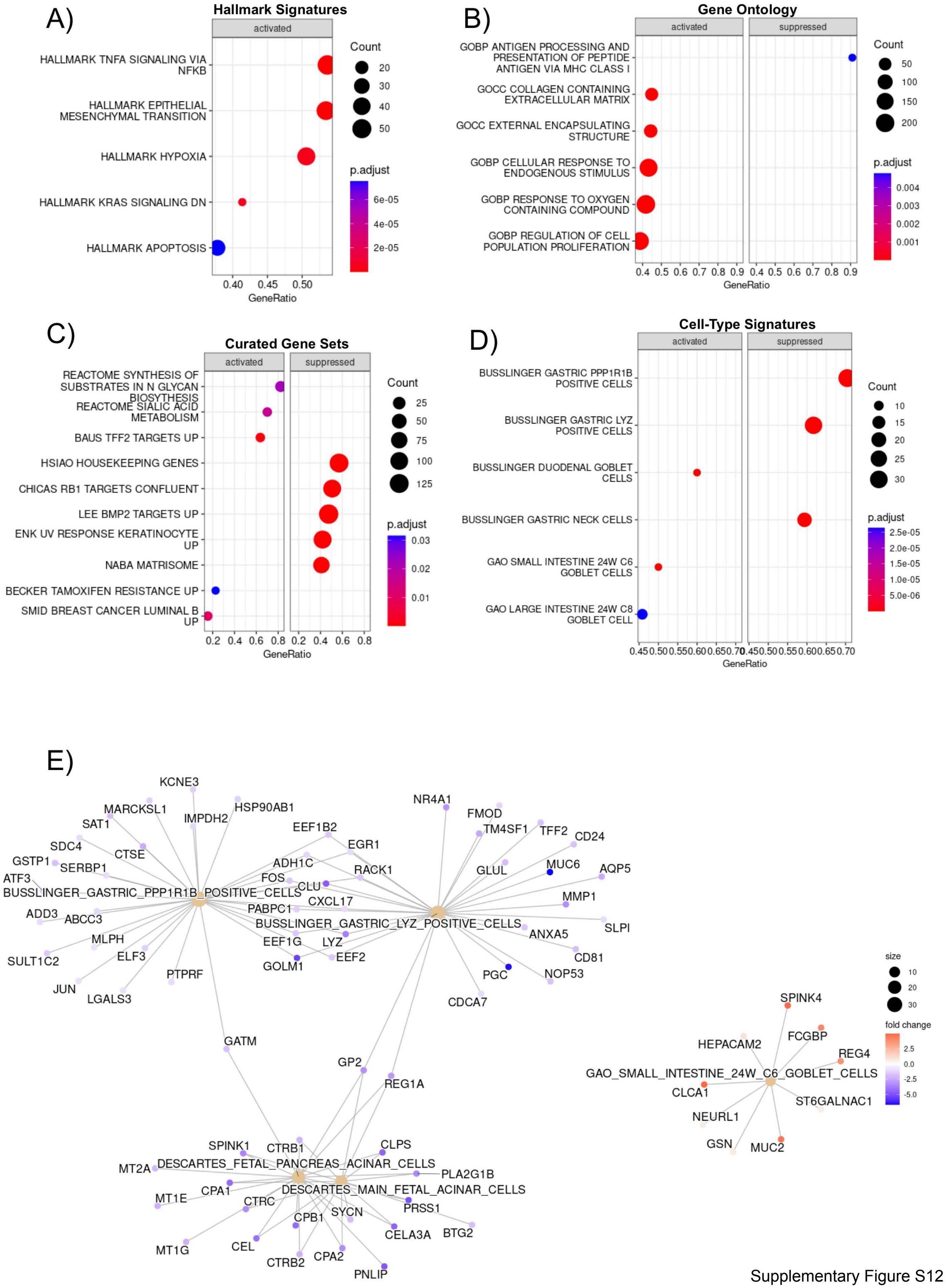

Supplementary Figure S12

Supplementary Figure S12. GSEA results of the comparison between HGD Intestinal IPMN and Borderline IPMN. A) Top Hallmark Cancer pathways activated in HGD Intestinal IPMN. The dot size represents the number of genes upregulated. Two-tailed GSEA corrected for multiple comparisons with FDR <0.05. B) Top five activated and suppressed gene ontology signature activated or suppressed in HGD Intestinal IPMN. The dot size represents the number of genes overexpressed or downregulated C) Top five activated and suppressed curated gene set activated or suppressed in HGD Intestinal IPMN. The circle size represents the number of genes overexpressed or downregulated. D) Top activated and suppressed cell type signature activated or suppressed in HGD Gastric IPMN. The circle size represents the number of genes overexpressed or downregulated. E) Network plot showing the expression of the cell type specific signatures. Abbreviations: Low-Grade-Dysplasia, LGD; High-Grade-Dysplasia, HGD; False Discovery Rate; FDR.Source data are provided as a Source Data file.

Supplementary Figure S13

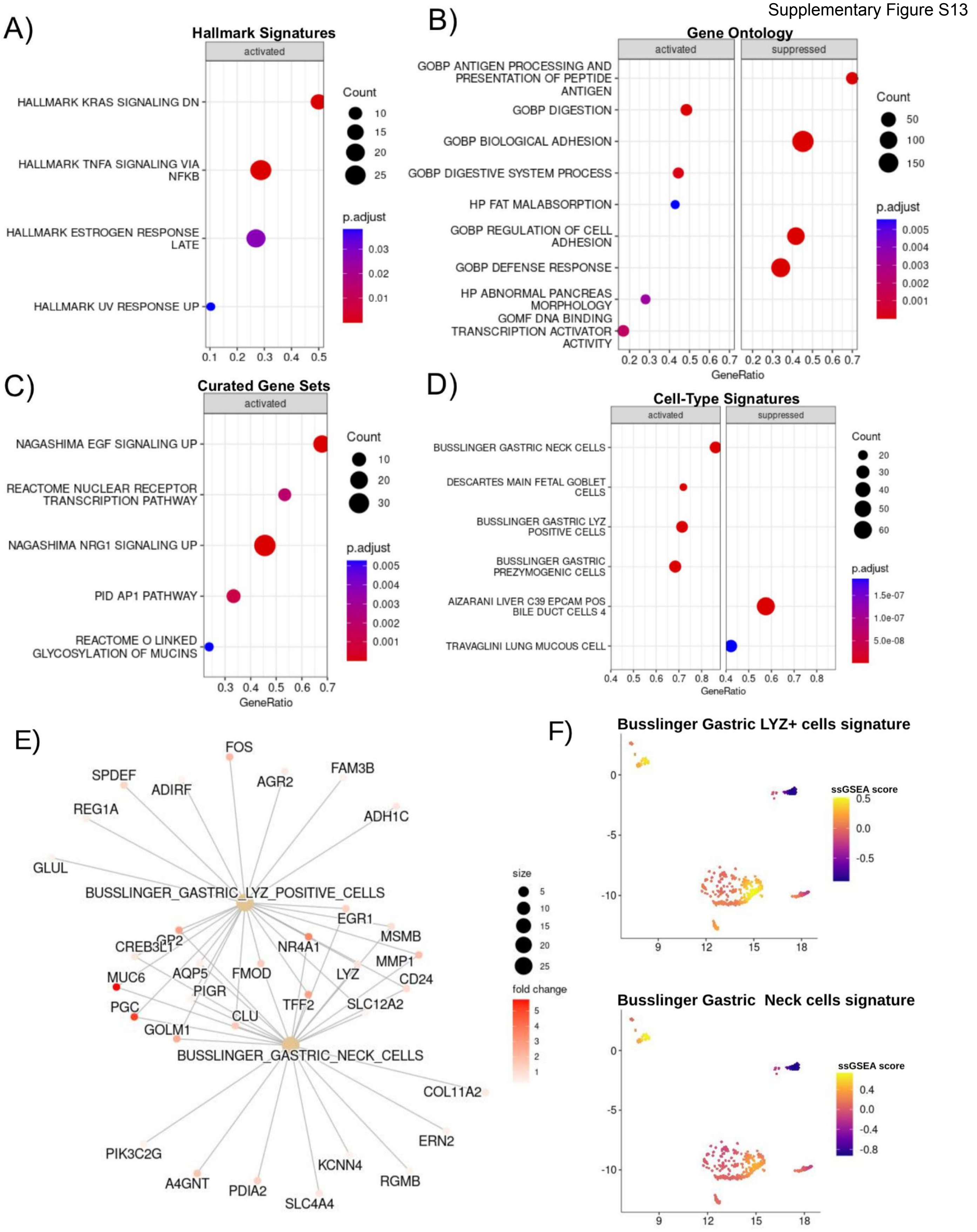

Supplementary Figure S13. GSEA results of the comparison between Borderline IPMN and LGD IPMN. A) Top Hallmark Cancer pathways activated in Borderline IPMN. The dot size represents the number of genes upregulated. Two-tailed GSEA corrected for multiple comparisons with FDR <0.05. B) Top five activated and suppressed gene ontology signature activated or suppressed in Borderline IPMN. The dot size represents the number of genes overexpressed or downregulated. C) Top five activated and suppressed curated gene set activated or suppressed in Borderline IPMN. The dot size represents the number of genes overexpressed or downregulated. D) Top activated and suppressed cell type signatures activated or suppressed in Borderline IPMN. The circle size represents the number of genes overexpressed or downregulated. E) Networkplot showing the expression of the cell type specific signatures. F) Feature plot showing the ssGSEA score for gastric cell signatures in IPMN clusters. Abbreviations: Low-Grade-Dysplasia, LGD; High-Grade-Dysplasia, HGD; False Discovery Rate; FDR.Source data are provided as a Source Data file.

Supplementary Note 2. Validation of IPMN Clusters via manual annotation excluding stroma shared spot and partially detached tissue.

To confirm the results obtained using an unbiased approach, we performed manual annotation of the IPMN clusters discarding the spots that were shared between IPMN and stromal cells and were occuring in the IPMN subjected to partial detachment. The figurebelow show the clusters that were manually annotated with an inset depicting the spot positions on the tissue, (Supplementary Information 2, Figure 1).

Following manual annotation, the spots underwent normalization and scaling using SCT transform. Differential expression analysis (DEA) was executed using the Findmarkers() function, configuring the DESeq2 method with a min.pct=0.3 (threefold higher than the default parameter). This adjustment aimed to filter out outlier genes that might be influenced by batch effects and consequently expressed aberrantly in only a few spots within the clusters with recommendations from Seurat developers (https://satijalab.org/seurat/ reference/findmarkers).

DEA between LGD IPMN and Borderline IPMN, as well as between HGD Gastric and Intestinal IPMN, yielded results consistent with the unbiased DEA analysis. This alignment is illustrated through Volcano plots and Cneplots, showcasing the expression of the primary signatures previously identified (please see Supplementary Information 2, Figure 2-4).

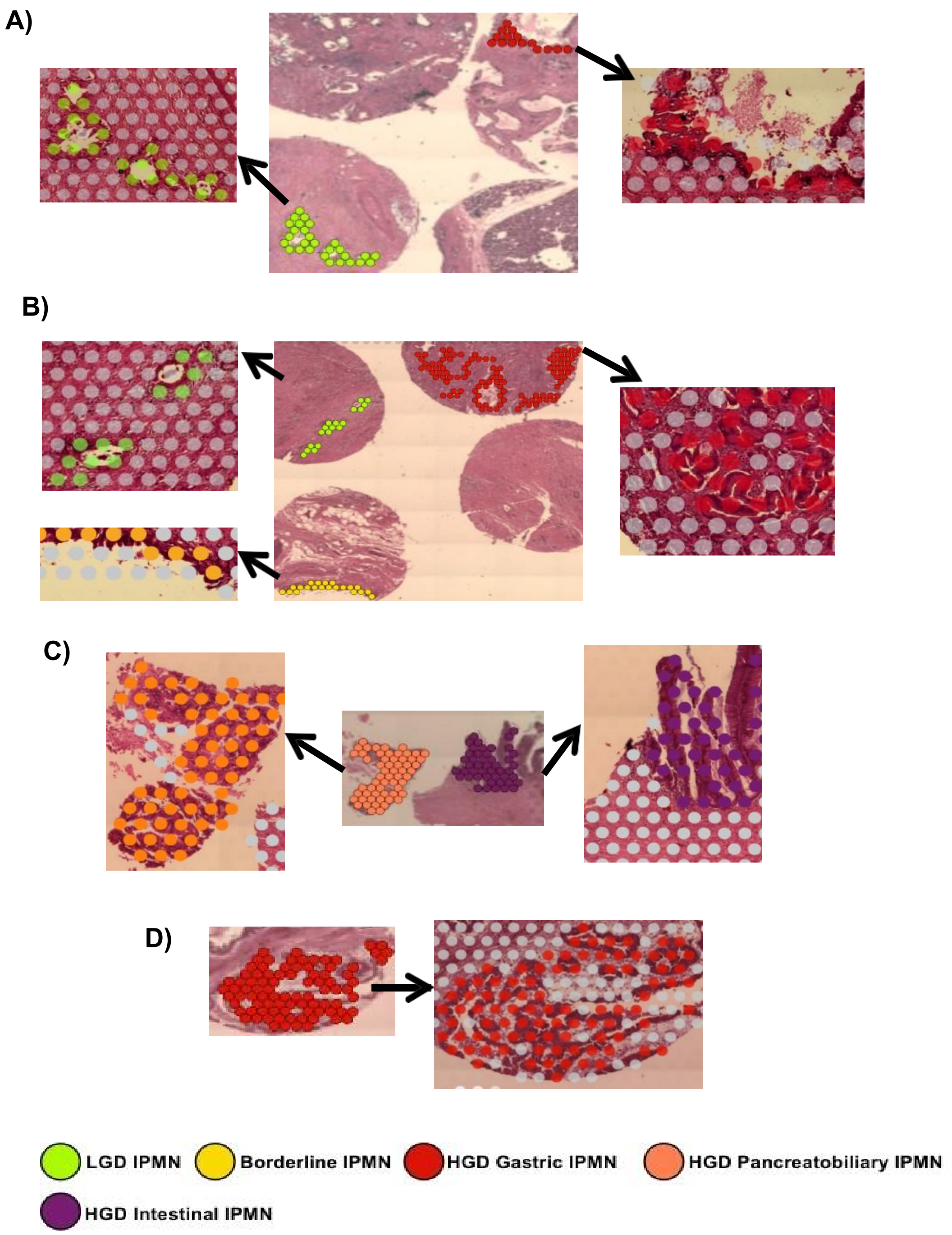

Supplementary Figure S14. Manual annotation of IPMN clusters. Spatial correlation between the manually annotated spots and histological figures in A) TMA1, B) TMA2, C) TMA3, and D) TMA4. Inlay shows thepathological association at greater magnification. Abbreviations: Low-Grade-Dysplasia, LGD; High-Grade-Dysplasia, HGD.

Supplementary Figure S15

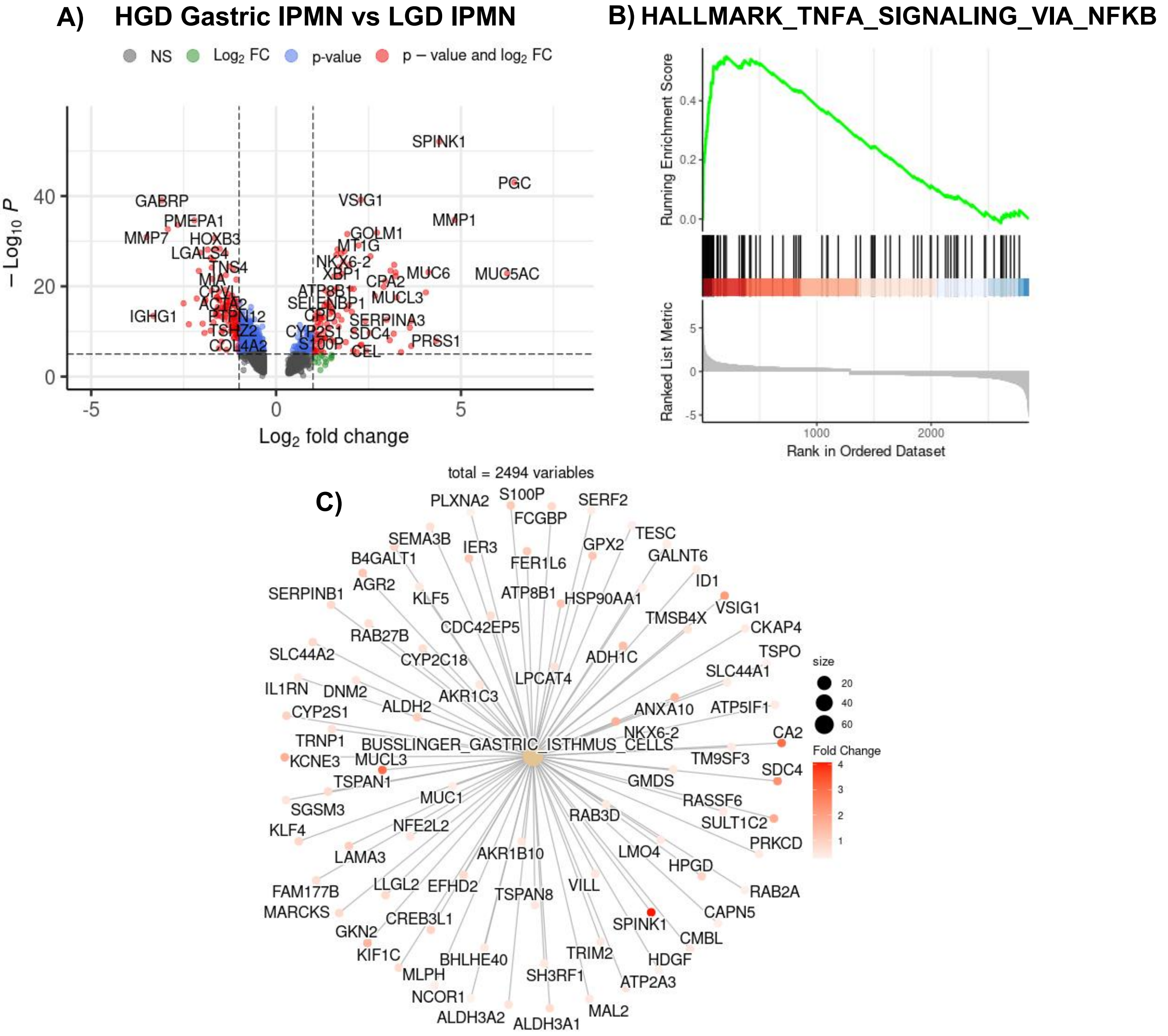

Supplementary Figure S15. DEA and GSEA results of the comparison between HGD Gastric IPMN and LGD IPMN. A) Volcanoplot showing the expression of the differentialexpressed genes between HGD Gastric IPMN and LGD IPMN. Log2 Fold Change < -1.5 and >1.5, FDR <0.05. B) GSEA plot showing the enrichment of HALLMARK_TNFA_SIGNALING_ VIA_NFKB signature in the upregulated genes of HGD Gastric IPMN.Two-tailed GSEA corrected for multiple comparisons with FDR <0.05 C) Networkplot showing the enrichment of the Busslinger Gastric Isthmus cells signature in HGD gastric IPMN. Color bar indicate the log2 Fold Change. Abbreviations: Low-Grade-Dysplasia, LGD; High-Grade-Dysplasia, HGD, False Discovery Rate, FDR.

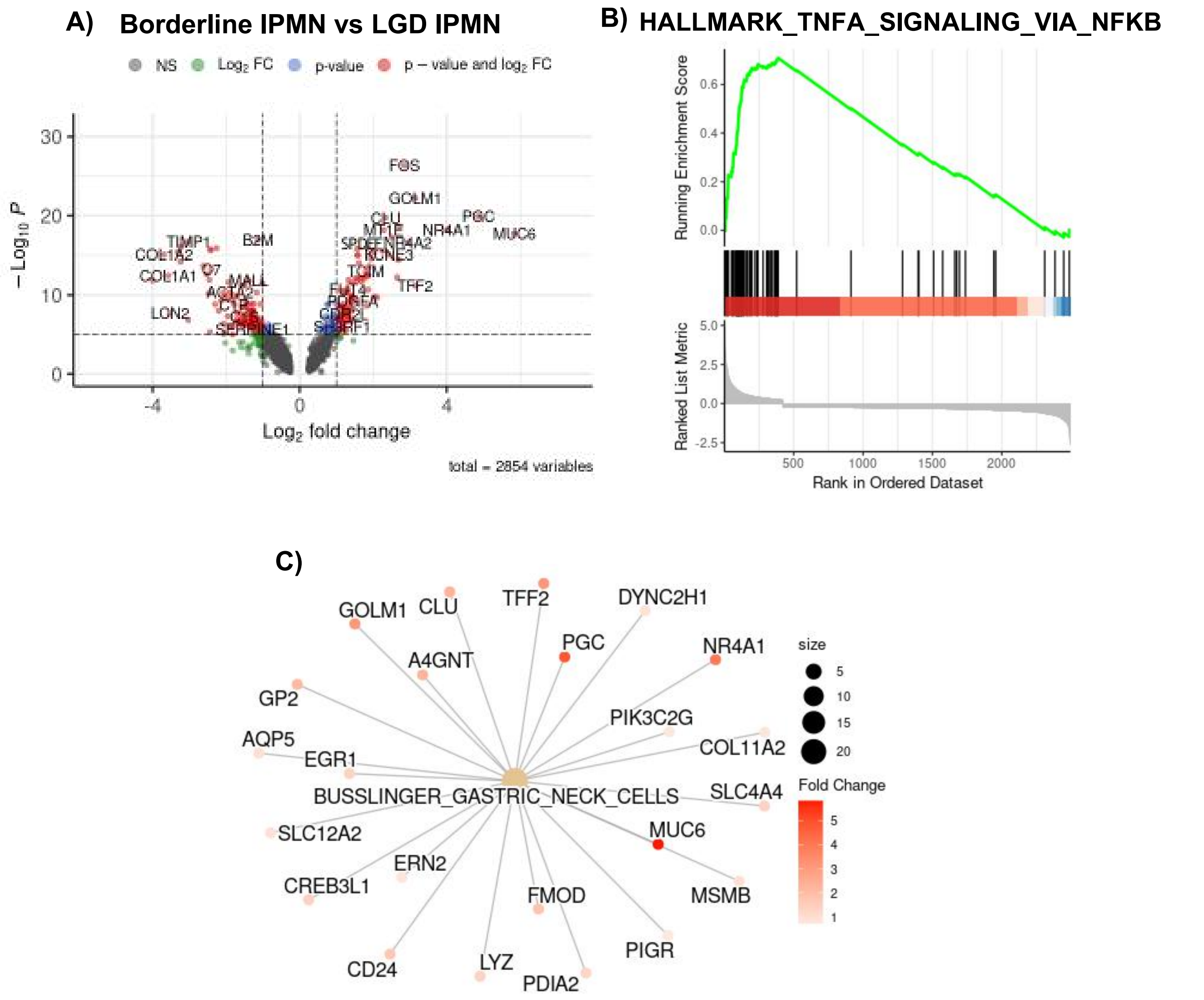

Supplementary Figure S16. DEA and GSEA results of the comparison between Borderline IPMN and LGD IPMN. A) Volcanoplot showing the expression of the differential expressed genes between Borderline IPMN and LGD IPMN. Log2 Fold Change < -1.5 and >1.5, FDR <0.05. B) GSEA plot showing the enrichment of HALLMARK_TNFA_SIGNALING_ VIA_NFKB signature in the upregulated genes of Borderline IPMN .Two-tailed GSEA corrected for multiple comparisons with FDR <0.05 C) Networkplot showing the enrichment of the Busslinger Gastric Neck cells signature in HGD gastric IPMN. Color bar indicate the log2 Fold Change. High-Grade-Dysplasia, HGD, False Discovery Rate, FDR.

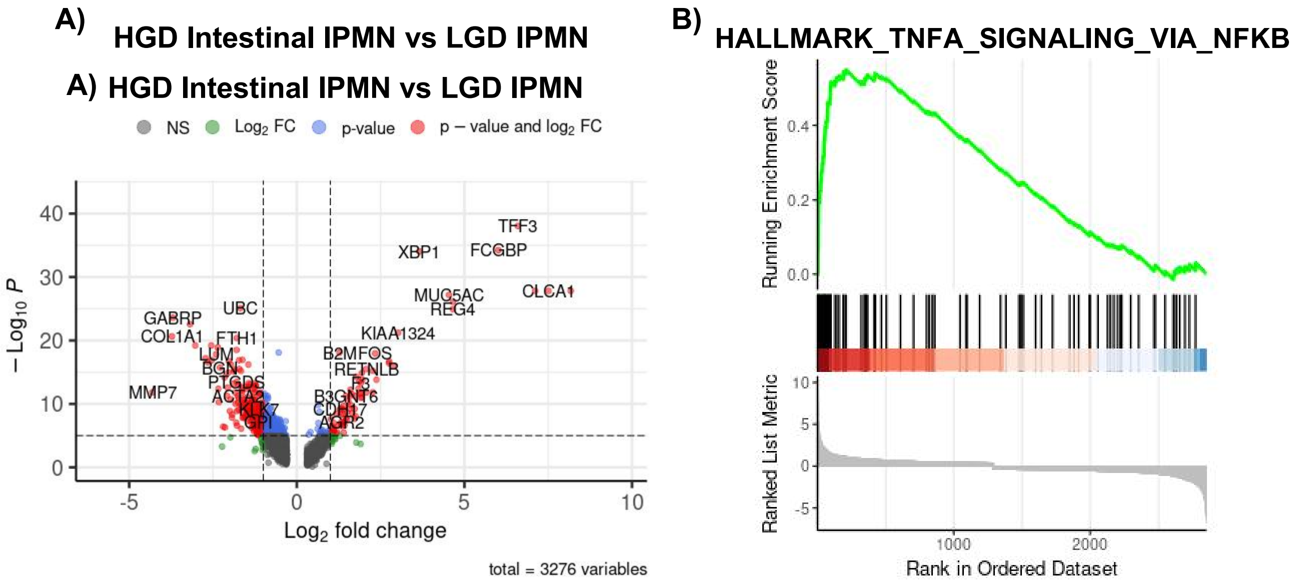

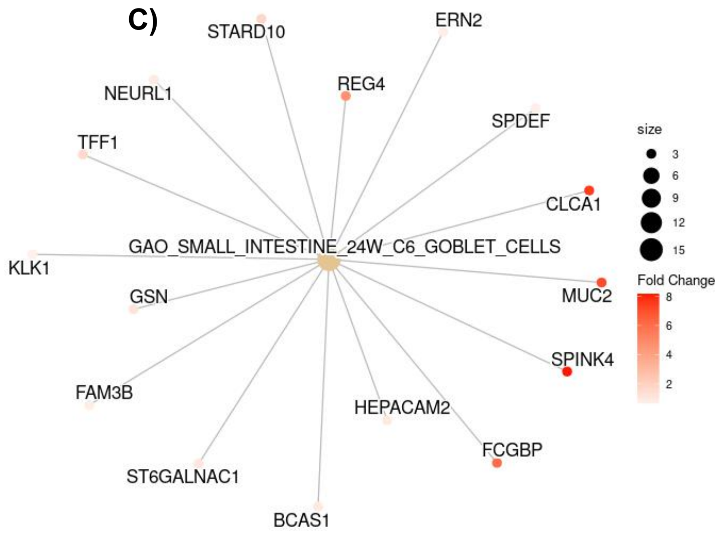

Supplementary Figure S17. DEA and GSEA results of the comparison between HGD Intestinal IPMN and LGD IPMN. A) Volcanoplot showing the expression of the differential expressed (DE) genes between HGD Intestinal IPMN and LGD IPMN. Log2 Fold Change < -1.5 and >1.5, FDR <0.05 B) GSEA plot showing the enrichment of HALLMARK_TNFA_SIGNALING_VIA_NFK signature in the upregulated genes of HGD Intestinal IPMN. Two-tailed GSEA corrected for multiple comparisons with FDR <0.05 C) Networkplot showing the enrichment of the GAO Small intestine 24W C6 Goblet cells signature in HGD Intestinal IPMN. Color bar indicate the log2 Fold Change. High-Grade-Dysplasia, HGD, False Discovery Rate, FDR.

Supplementary Figure S18

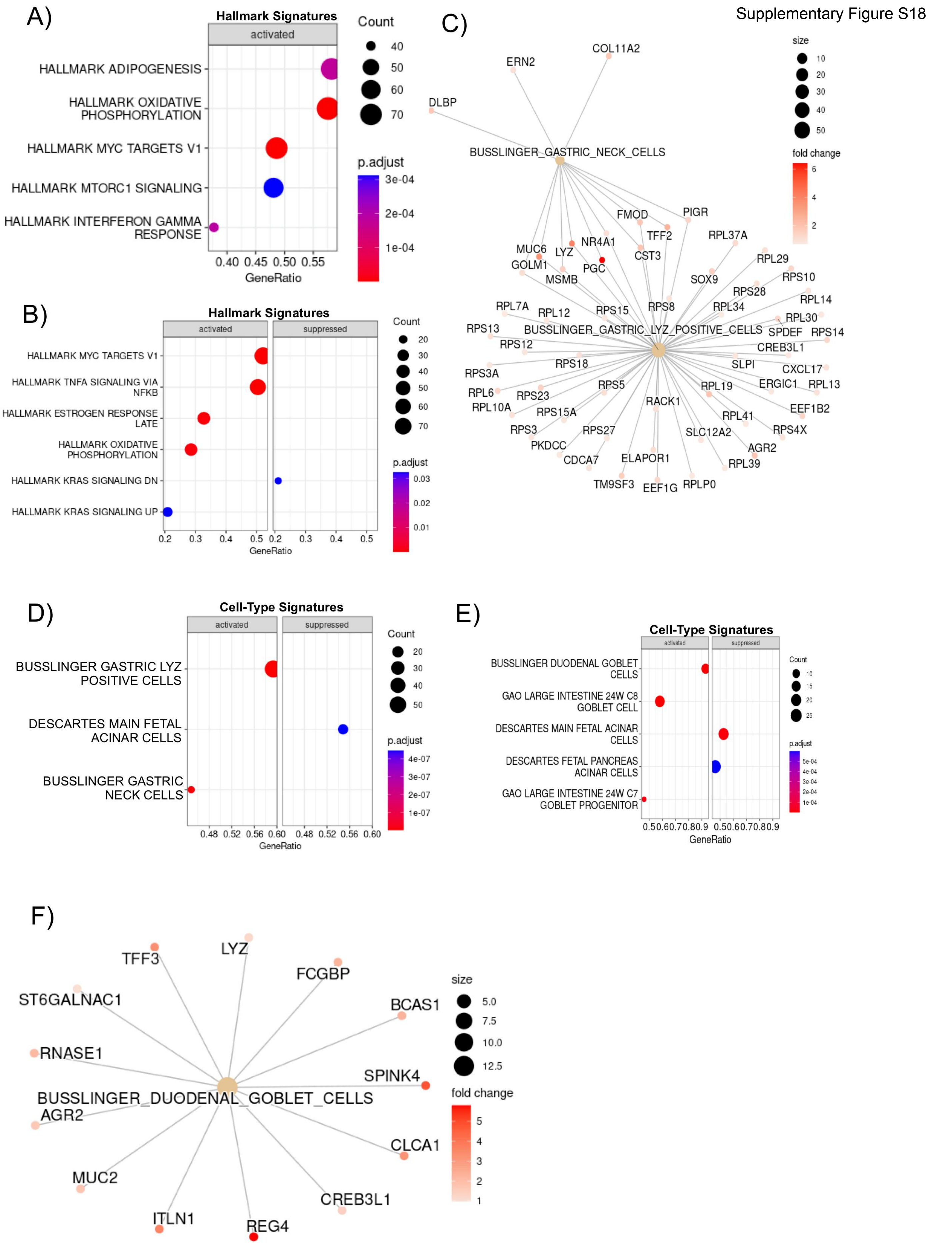

Supplementary Figure S18. GSEA results from DEA (GeoMx ST Data). A) and B) Dotplots showing the top Hallmark Cancer pathways activated in Borderline and Intestinal IPMN when compared to LGD IPMN. C) and D) Gastric Neck Cell signature upregulated in Borderline IPMN. E) Cell type signatures activated in HGD Intestinal IPMN when compared to LGD IPMN. F) Duodenal goblet cell signatures activated in HGD Intestinal IPMN**.** Two-tailed GSEA corrected for multiple comparisons with FDR <0.05. Abbreviations: Low-Grade-Dysplasia, LGD; High-Grade-Dysplasia, HGD; False Discovery Rate; FDR.Source data are provided as a Source Data file.

	LGD	BR	HGD GASTRIC	HGD INTESTINAL	HGD PANCREATOBILIARY
HOXB3	1	1	0	0	0
SPDEF	0	1	0	1	0
NKX6-2	0	1	1	0	0

EXPRESSION	
1	YES
0	NO

EXPRESSION LEVELS	
	LOW
	HIGH

Supplementary Table S2. Example Table for the precise assessment of IPMN subtype and grading. The table showed the presence/1 absence/0 of genes validated in Immunofluorescence analysis and their expression levels (Dark Grey /High, Grey/Low). Abbreviations: Low-Grade-Dysplasia, LGD; Borderline, BR; High-Grade-Dysplasia HGD.

Supplementary Methods. Additional methods for Bioinformatics analyses.

System Parameters

All analyses were performed on a local machine with 32 cores and 128Gb RAM running Ubuntu Linux 22.04 LTS. Secondary analyses were performed in R v4.2.2 with RStudio build 353, and in python with anaconda v2022.05.

Visium

Fastq files Processing and Quality Control

H&E stain stitched images (20X) of each TMA were acquired with EVOS FL Auto II.The images were manually aligned with Loupe Browser v5.2 (10X Genomics) to the fiducial frames to match tissue images with spot positions. Fastq files were processed with Space Ranger 1.3.1 (10X genomics) using recommended parameters for FFPE samples using human transcriptome provided by 10X Genomics (GRCh38-2020-A), the H&E images, and the manual alignment files for each capture area. SpaceRanger generated a series of output files to be analyzed for secondary analysis and a summaryfor all key quality control parameters for sequencing, gene mapping, ans spot coverage (https://support.10xgenomics.com/spatial-gene-expression/software/pipeline s/latest/output/summary). All capture areas passed the quality control checks.

Dataset Integration and Clustering

SpaceRanger outputs for each TMA (filtered count files, tissue position lists, high-resolution images, and scale factor files) were loaded in R with the package STUtility 1.1.1 subsequently transformed and merged in a Seurat object (Seurat v4.3.0). A standard Seurat (4.3.0.1) workflow was followed with minor modifications for spatial transcriptomics. Variable features were found with FindVariableFeatures() function using variance stabilizing transformation (vst) method. The Default Seurat dataset integration function (SCTransform) in conjunction with Harmony 1.1.0 algorithm (used with default parameters) was performed to remove batch effect and tointegrate the datasets. Uniform Manifold Approximation and Projection (UMAP) dimensional reduction technique was performed with RunUMAP() on the first 30 dimension of the Harmony reduction (parameter reduction=”harmony”) Nearest-neighbor graph construction was performed with FindNeighbors() function taking in consideration only the 30 dimensions of Harmony reduction and number of neighbors of six (Visium spots may be approximated to a hexagon). Clustering was performed with Seurat Findclusters() function using the leiden method with a resolution of 0.85 after testing several parameters to avoid sub-optimal clustering

Cell-type Inference and Cluster annotation

The main markers of each cluster were found with Findmarkers() function withmin.pct parameter of 0.3, using the DESeq2 (1.40.2) method. Main markers for IPMN clusters were visualized with a Violinplot of log2 expression using standard Seurat visualization functions. Cell-type inference was performed using two different R packages Azimuth and AUCell 1.22.0. The RunAzimuth() function was used to perform a reference-based mapping using Azimuth pancreas reference to evaluate the composition of the various pancreatic tissues present in the samples. However since the resolution of Visium is of 55μm most of the clusters were not pure pancreatic cells but an admixture of pancreatic and other stromal and TME cells. To evaluate the presence of such cells in the tissue we used the UCell package to calculate the module scores for the signatures characterizing the normal and tumor-associated stromal cells such as stellate cells, cancer associated fibroblasts, dendritic cells, and macrophages and other. Cell-type specific signatures were downloaded from Panglao database, and used as input for the AddModuleScore_UCell(). Only the spots with a score > 0.6 were considered to be enriched for a specific cell type. For some stromal cluster we could not infer a specific cell composition and we therefore we named accordingly to the main markers composition and histological features (i.e Pancreatic stroma 1-3, Fibrotic tissue 1-2, Acinar tissue 1-3, and Immune rich stroma 1-2).

Differential expression analysis (DEA), Gene set enrichment analysis (GSEA), and Transcription Factor Activity.

We performed DEA between IPMN clusters using the Seurat Findmarkers() function using the DESeq2 method. The R package clusteRprofiler 4.8.3 was used for GSEA using the DEA output filtering out all the genes with a fold change comprised between -1 and 1 and a p.adj > 0.1 interrogating the MsigDB gene set collections (Hallmark cancer, Gene Ontology, Curated gene sets, and Cell type signature gene sets). Only the pathway with a normalized enrichment score (NES) < -1 and > 1 with a FDR < 0.05 were considered. ClusteRprofiler visualization plots were used for figures to show the main enriched pathways and gene networks. Single sample GSEA (ssGSEA) was also scored for each MsigDB gene sets found deregulated using the R package escape 1.10.0. Transcription Factor activity was assessed with SCENIC(pySCENIC 0.12.1) with default parameters.

Spatial Trajectory Inference

SpaceRanger outputs for TMA 1 and 2 (filtered count files, tissue position lists, high-resolution images, and scale factor files) were analyzed with STlearn package 0.4.0 and Scanpy Scanpy 1.9.4. We followed standard workflow (https://stlearn. readthedocs.io/en/latest/tutorials.html) with minor modifications.

PCA was calculated with sc.pp.pca() using Halko randomized algorithm (svd_solver= "randomized"). Dataset integration was performed using the scanpy regress_out function and Harmony using standard parameters. Neighborhood graph was calculated with sc.pp.neighbors() using the 30 dimensions of Harmony reductionusing neighbors parameter equal to six. Leiden Clustering was performed with sc.tl.leiden(). We used the following method to infer spatial trajectory between Borderline and HGD Gastric IPMN. To infer spatial trajectory the root spot of the cluster was choosen to be in the end/begin of a cluster in UMAP space. The opportuneroot was set with the function st.spatial.trajectory.set_root() for Borderline IPMN cluster. We then run the global level of pseudo-time-space (PSTS) method to reconstruct the spatial trajectory between Borderline IPMN and HGD Gastric IPMN clusters. St.spatial.trajectory.detect_transition_markers_clades() function was used to identify the transition markers that positive correlated with trajectory (Spearman > 0.4). Diffusion Pseudotime with Scanpy was also calculated to to show the associaton of transcription factor markers with trajectory.

GeoMx

Fastq files processing and QC

Fastq files were converted in DCC (Digital Count Conversion) files with NanoString GeoMx® NGS Pipeline 2.0.0 on DRAGEN v4.1.(Illumina). DCC files were analyzed in R using the GeomxToools 3.4.0 and GeoMxWorkflows 1.2.0.

All the ROI that showed poor sequencing (saturation <45%) , high signal-to-noise ratio were discarded from the secondary analysis.

Seurat Object Conversion and Analyses

Before Converting GeoMx data in a Seurat object gene counts were normalized with Geomxtools function normalize() with the negative control normalization method. After conversion the GeoMx seurat object was analyzed following standard workflow with minor modifications. Data from TMA 5 and 6 were integrated using Harmony. After, leiden clustering was performed with the first 15 dimensions of Harmony reduction. ROI were annotated according to histological features in LGD, Borderline, HGD Gastric and HGD Intestinal IPMN. Findmarkers() function was used to validate IPMN markers and perform DEA between IPMN groups using the Seurat function FindMarkers() with the DESeq2 method. GSEA was performed as described above in the Visium analysis section
