# Supplementary material for: Identification of spatially-resolved markers of malignant transformation in Intraductal Papillary Mucinous Neoplasms
Source: Nat Commun. 2024 Mar 29;15:2764. doi: 10.1038/s41467-024-46994-2 (PMC10980816; doi:10.1038/s41467-024-46994-2)
Supplement: Supplementary file 3 — Reporting Summary [file 41467_2024_46994_MOESM3_ESM.pdf]

nature portfolio

Corresponding author(s): Carmine Carbone

Last updated by author(s): Feb 16, 2024

Reporting Summary

Nature Portfolio wishes to improve the reproducibility of the work that we publish. This form provides structure for consistency and transparency in reporting. For further information on Nature Portfolio policies, see our Editorial Policies and the Editorial Policy Checklist.

Statistics

For all statistical analyses, confirm that the following items are present in the figure legend, table legend, main text, or Methods section.

n/a	Confirmed	
☐	☒	The exact sample size (n) for each experimental group/condition, given as a discrete number and unit of measurement
☐	☒	A statement on whether measurements were taken from distinct samples or whether the same sample was measured repeatedly
☐	☒	The statistical test(s) used AND whether they are one- or two-sided<br>*Only common tests should be described solely by name; describe more complex techniques in the Methods section.*
☐	☒	A description of all covariates tested
☐	☒	A description of any assumptions or corrections, such as tests of normality and adjustment for multiple comparisons
☐	☒	A full description of the statistical parameters including central tendency (e.g. means) or other basic estimates (e.g. regression coefficient) AND variation (e.g. standard deviation) or associated estimates of uncertainty (e.g. confidence intervals)
☐	☒	For null hypothesis testing, the test statistic (e.g. F, t, r) with confidence intervals, effect sizes, degrees of freedom and P value noted<br>*Give P values as exact values whenever suitable.*
☒	☐	For Bayesian analysis, information on the choice of priors and Markov chain Monte Carlo settings
☐	☒	For hierarchical and complex designs, identification of the appropriate level for tests and full reporting of outcomes
☐	☒	Estimates of effect sizes (e.g. Cohen's d, Pearson's r), indicating how they were calculated

Our web collection on statistics for biologists contains articles on many of the points above.

Software and code

Policy information about availability of computer code

Data collection	TMA build: Galileo TMA CK4600<br>FFPE sections: Leica SM2000R<br>Visium: Thermofisher EVOS FL Auto2(Imaging), Sequencing Novaseq6000 Illumina<br>GeoMx: GeoMx Digital Spatial Profiler, Sequencing Novaseq6000 Illumina<br>Multiplex Immunofluorescence: Akoya Phenoimager
Data analysis	Space Ranger 1.3.1<br>STUtility 1.1.1<br>Seurat 4.3.0.1<br>AUCell 1.22.0<br>clusteRprofiler 4.8.3<br>escape 1.10.0<br>pySCENIC 0.12.1<br>GeoMxWorkflows 1.2.0<br>DRAGEN v4.1<br>NanoString GeoMx® NGS Pipeline 2.0.0<br>GeoMxTools 3.4.0<br>stLearn 0.4.0<br>QuPath 0.4.2<br>R v4.2.2<br>RStudio build 353

anaconda v2022.05
Loupe Browser v5.2
Harmony 1.1.0
DESeq2 (1.40.2)

For manuscripts utilizing custom algorithms or software that are central to the research but not yet described in published literature, software must be made available to editors and reviewers. We strongly encourage code deposition in a community repository (e.g. GitHub). See the Nature Portfolio guidelines for submitting code & software for further information.

Data

Policy information about availability of data

All manuscripts must include a data availability statement. This statement should provide the following information, where applicable:

- Accession codes, unique identifiers, or web links for publicly available datasets
- A description of any restrictions on data availability
- For clinical datasets or third party data, please ensure that the statement adheres to our policy

The Spatial Transcriptomics data generated in this study have been deposited in the GEO database under accession codes: GSE229877 [https://www.ncbi.nlm.nih.gov/geo/query/acc.cgi?acc=GSE229877] (Visium raw and processed data) and GSE229752 [http://www.ncbi.nlm.nih.gov/geo/query/acc.cgi?acc=GSE229752] (GeoMx raw and processed data) TruSight500 raw data is available on Sequenced Read Archive (SRA) BioProject SRA number: PRJNA1013719 [https://www.ncbi.nlm.nih.gov/bioproject/PRJNA1013719]. Source data are provided with this paper.

Research involving human participants, their data, or biological material

Policy information about studies with human participants or human data. See also policy information about sex, gender (identity/presentation), and sexual orientation and race, ethnicity and racism.

Reporting on sex and gender	This study has no sex and/or gender-based analyses, and we do not collect this information.
Reporting on race, ethnicity, or other socially relevant groupings	No data about race, ethnicity, or other socially relevant groupings was collected for this study
Population characteristics	The Visium cohort consisted of FFPE from 14 patients, including four with low-grade IPMN, 9 high-grade IPMN characterized by HGD (of whom 4 had IPMN-associated PDAC) and PDAC-associated normal duct (n=1). Low-grade IPMN included three LGD lesions and one Borderline IPMN with an intermediate grade of dysplasia. High-grade IPMN included invasive lesions characterized by a high-grade of dysplasia representing the three morphotypes: Gastric (n=5), Intestinal (n=3) and Pancreatobiliary (n=1).<br><br>The GeoMx validation cohort was composed of 101 tissue cores from 61 patients. IPMN samples were obtained from the APGI, as part of the International Cancer Genome Consortium (ICGC). Two TMAs (named TMA5 and TMA6) with 101 clinically annotated IPMN cores were collected including LGD, Borderline, and HGD IPMN of different morphology. These TMAs were used for ST with the Nanostring GeoMx Digital Spatial Profiler.<br><br>For markers validation, we conducted multiplex immunofluorescence (Multiplex-IF) analysis using a set of archival IPMN samples, comprising 16 specimens in total. This cohort includes 4 low-grade cases, 4 HGD Gastric subtype cases, 4 HGD Intestinal subtype cases, and 4 HGD Pancreatobiliary subtype cases. Additionally, 2 samples of normal ducts were included in the analysis.
Recruitment	The participants were selected based on the pathological diagnosis results and the H & E staining of tissue, without considering factors such as age or gender. No biases are involved in the participants selection.
Ethics oversight	Clinical samples collection was approved by the local ethics committee (Fondazione Policlinico Gemelli IRCCS, Ethical Committee approval Prot. Gen. 3536) and followed EU regulations. All partecipants provided written informed consent for sample collection and subsequent analyses publication.

Note that full information on the approval of the study protocol must also be provided in the manuscript.

Field-specific reporting

Please select the one below that is the best fit for your research. If you are not sure, read the appropriate sections before making your selection.

☒ Life sciences ☐ Behavioural & social sciences ☐ Ecological, evolutionary & environmental sciences

For a reference copy of the document with all sections, see nature.com/documents/nr-reporting-summary-flat.pdf

Life sciences study design

All studies must disclose on these points even when the disclosure is negative.

Sample size	IPMN are rare tumors so we tried to analyze as many samples as we could for each type of analyses.<br>We referred to previous publications to estimate the number of samples to analyze rather than using sample size calculations.<br>However for GeoMx analyses we used strict guidelines for ROI selections (please see MAN-10154-01-GeoMx-DSP-Data-Analysis-User-Manual for more details). In particular we used at least 6 ROIs from different samples for each condition, and we selected ROI with above 100 nuclei, as suggested in the aforementioned manual.
Data exclusions	23 GeoMx ROI that failed at QC step were excluded from the analyses.
Replication	Information were included in the figure legends.
Randomization	Randomization was not applicabile to this study as we wanted to identify the markers that distinguished the different groups of IPMN.
Blinding	The operator that performed Multiplex IF image analysis was blinded to the diagnosis. Moreover, IPMN histopathological features were confirmed by two expert pathologists in blind.

Reporting for specific materials, systems and methods

We require information from authors about some types of materials, experimental systems and methods used in many studies. Here, indicate whether each material, system or method listed is relevant to your study. If you are not sure if a list item applies to your research, read the appropriate section before selecting a response.

Materials & experimental systems

n/a	Involved in the study	
☐	☒	Antibodies
☒	☐	Eukaryotic cell lines
☒	☐	Palaeontology and archaeology
☒	☐	Animals and other organisms
☒	☐	Clinical data
☒	☐	Dual use research of concern
☒	☐	Plants

Methods

n/a	Involved in the study	
☒	☐	ChIP-seq
☒	☐	Flow cytometry
☒	☐	MRI-based neuroimaging

Antibodies

Antibodies used	Primary antibodies: HOXB3 (PA5-103890, Thermo Fisher Scientific), SPDEF (ab220776, ABCAM), NKX6-2 (ABN-1455, MERCK), PanCK (67306S, CellSignaling).<br><br>Secondary Antibodies: the Opal 6-Plex Detection Kit (NEL821001KT,Akoya Biosciences).
Validation	Before proceeding, optimal staining conditions for each marker were determined using monoplex stained slides from a positive control for each antibody. Protein Atlas (www.proteinatlas.org) was used to identify the positive control tissue that express high levels of each protein.<br>HOXB3 (PA5-103890): Polyclonal Rabbit / IgG, reactive with human, We tested the antibody for IF first on both IPMN and on testis samples. Final dilution 1:100<br>SPDEF (ab220776): Polyclonal Rabbit / IgG, reactive with human, We tested the antibody for IF first on both IPMN and on salivary gland samples. Final dilution 1:400<br>NKX6-2 (ABN-1455): Polyclonal Rabbit / IgG, reactive with human, We tested the antibody for IF first on both IPMN and on spinal cord samples. Final dilution 1:1000<br>PanCK (67306S):Polyclonal Mouse/ IgG reactive with human, We tested the antibody for IF first on both IPMN and on lung and colon cancer samples. Final dilution 1:1000<br><br>For Multiplex IF we used the Opal 6-Plex Detection Kit (NEL821001KT) using the oppurtune secondary antibody for each primary.
